# Religion and Chloroform

**Published:** 1879-04

**Authors:** 


Religion and Chloroform.—Dr. B. W. Richardson lately gave a Sunday afternoon lecture in London on "Anæsthetic Sleep and the Temporary Abolition of Pain." He remarked that the credit of having introduced chloroform belonged to the late Sir James Simpson, of Edinburgh. Its introduction and application were objected to on religious grounds, some people contending that man, according to Scripture, should endure pain and trouble throughout life. Sir James Simpson threw the scriptural argument back upon those who used it by saying that when the first man had an operation performed upon him he was put in a deep sleep, and knew nothing of the time when the rib was taken from him.—*Journal of Chemistry.*

